# Robotic-Assisted Spinal Instrumentation from C1 to S1 Experience of a UK Neurosurgical Tertiary Referral Centre

**DOI:** 10.3390/jcm15155905

**Published:** 2026-07-29

**Authors:** Asfand Baig Mirza, Wajiha Rauf, Ibrahim Muhyiddin Muhammad, Feras Fayez, Ariadni Georgiannakis, Amisha Vastani, Varinder Singh Alg, Anjum Qureshi, Bhaskar Thakur, Babak Arvin, Ahmed-Ramadan Sadek, Taofiq Desmond Sanusi

**Affiliations:** 1 Department of Neurosurgery, Queen’s Hospital, Romford, Barking, Havering and Redbridge University Hospitals NHS Trust, Romford RM7 0AG, UK; asfand.mirza@nhs.net (A.B.M.); wajiha.rauf@nhs.net (W.R.); varinder.alg@nhs.net (V.S.A.); anjum.qureshi1@nhs.net (A.Q.); bhaskar.thakur@nhs.net (B.T.); arvin.babak@nhs.net (B.A.); ahmed-ramadan.sadek@nhs.net (A.-R.S.); 2 Barts and The London School of Medicine and Dentistry, London E1 2AD, UK; i.muhammad@smd21.qmul.ac.uk (I.M.M.); a.georgiannakis@smd20.qmul.ac.uk (A.G.); 3 National Hospital for Neurology and Neurosurgery, Queen Square, London WC1N 3BG, UK; feras.fayez@nhs.net; 4 Department of Neurosurgery, King’s College Hospital NHS Foundation Trust, London SE5 9RS, UK; a.vastani@nhs.net

**Keywords:** robotic spine surgery, learning curve, CUSUM, fluoroscopy, emergency spinal surgery, UK NHS, C1 to S2, neurosurgery

## Abstract

**Background:** Robotic-assisted spinal instrumentation is increasingly used to support implant placement, reduce radiation exposure, and improve operative workflow. However, UK NHS data describing early programme experience across elective and emergency practice remain limited. This study aimed to evaluate the feasibility, safety profile, and early learning curve of robotic-assisted spinal instrumentation in an unselected UK NHS cohort. **Methods:** A retrospective, single-centre, single-arm observational cohort study was conducted, including the first 50 consecutive patients treated from programme inception between June 2024 and January 2026. No exclusion criteria were applied, and each record represented one patient and one operation. Missing data were not imputed, and denominators were reported per variable. Learning curve effects were assessed using Spearman correlation and cumulative sum analysis. Wilson 95% confidence intervals were reported for complication rates. **Results:** The mean age was 61.9 years; 50% were male; mean body mass index was 29.0; and median Charlson Comorbidity Index was 3. Indications were degenerative disease in 74%, trauma in 24%, and deformity in 2%. Emergency admissions accounted for 18/50 cases. Robot-specific adverse events included abandonment or conversion in 2/50 cases and system malfunction in 1/50. Surgical complications occurred in 7/50 patients. On surgeon-reviewed routine post-operative imaging, no screw malpositions required revision and no durotomies or vascular injuries were recorded; this represents a clinical revision rate rather than a formal radiological measure of screw accuracy, for which blinded Gertzbein–Robbins grading was not performed. Cumulative sum analysis suggested a potential fluoroscopy change point around case 30, with the median fluoroscopy events falling from 151 to 16 thereafter. Emergency cases involved more instrumented levels, longer operative times, and more open surgery than elective cases. **Conclusions:** Robotic-assisted spinal instrumentation was feasible across a diverse UK NHS caseload from C1 to S2, with a low observed complication rate. The findings suggest a fluoroscopy learning curve threshold around case 30. Larger comparative studies with formal radiological accuracy assessment are required.

## 1. Introduction

Robotic-assisted spinal instrumentation is transforming the landscape of spine surgery globally. Driven by the recognised limitations of conventional pedicle screw placement—with technique-dependent malposition rates of 10–40% across 51,161 screws in a comprehensive systematic review [1]—robotic guidance offers surgeons reproducible trajectory planning, real-time navigation integration, and a measurable reduction in theatre staff radiation exposure [2,3]. As the National Health Service (NHS) embarks on its most ambitious expansion of robotic surgery to date, projecting 500,000 robot-assisted procedures annually by 2035 [4], spine surgery represents one of the most technically demanding domains in which this technology can be applied [5].

The United Kingdom (UK) has been a relative latecomer to published robotic spine series. While several abstract reports have described early experiences with robotic spinal surgery in UK centres [6], to our knowledge, no comprehensive, peer-reviewed cohort study of robotic spinal instrumentation from a UK NHS unit has been published. The applicability of these experiences to the NHS—with its high emergency throughput, diverse spinal pathology, and established navigation ecosystems—has not been directly evaluated.

The present series describes our initial experience with robotic-assisted spinal instrumentation at Queen’s Hospital, Romford, Barking, Havering and Redbridge University Hospitals (BHRUT), covering the first 50 consecutive cases from programme inception. The cohort spans the complete spinal axis from C1 to S2 across elective, emergency, trauma, degenerative, and deformity pathology, a breadth of unselected real-world presentations not previously characterised in a published UK series.

The programme was established by the senior author (TDS) with structured robotic onboarding, integration of intraoperative CT navigation, and manufacturer proctoring for initial cases. Our objectives are as follows: (1) to describe the cohort characteristics and demonstrate the breadth of spinal pathology managed robotically; (2) to report a cumulative sum (CUSUM)-based fluoroscopy learning curve; (3) to compare operative variables between elective and emergency admissions; and (4) to characterise observed complications with 95% confidence intervals. This report is explicitly framed as an early experience series. No control group was included; the outcomes cannot be attributed to the robotic device versus existing navigation infrastructure or surgeon skill.

## 2. Materials and Methods

### 2.1. Study Design and Setting

This was a single-centre, retrospective cohort study conducted in the Department of Neurosurgery at Queen’s Hospital, Romford, BHRUT, UK. Data were extracted and analysed retrospectively from existing clinical records from programme inception (June 2024) to January 2026, according to a predefined protocol. The data sources included electronic health records, operative notes, imaging records, theatre documentation, and follow-up documentation. No study-specific investigations, visits, or interventions were mandated; patient management and documentation followed routine clinical practice, and data were extracted after the relevant clinical encounters had taken place. The study was registered as a clinical governance audit under BHRUT Research and Development oversight and was conducted and reported in accordance with STrengthening the Reporting of OBservational studies in Epidemiology (STROBE) guidelines for observational cohort studies. Given the retrospective nature of the study and the use of anonymised routinely collected clinical data, the requirement for individual informed consent given as part of standard clinical consent practice within BHRUT and the wider UK National Health Service, patients are informed that anonymised clinical information may be used for audit, quality improvement, teaching, and educational publications as part of the consent process.

### 2.2. Participants and Unit of Analysis

All 50 consecutive patients undergoing robotic-assisted spinal instrumentation from programme inception to the 50th case were included; each record represents one patient and one operation. No exclusion criteria were applied; the cohort is entirely unselected and consecutive, including emergency out-of-hours presentations. The operating consultant (TDS) used the robotic system when intraoperative navigation infrastructure was available and clinically appropriate. Cases requiring immediate unplanned surgery before navigation was available were managed conventionally and are not captured in this retrospective robotic-assisted cohort.

### 2.3. Robotic System and Operative Workflow

**Figure 1:**
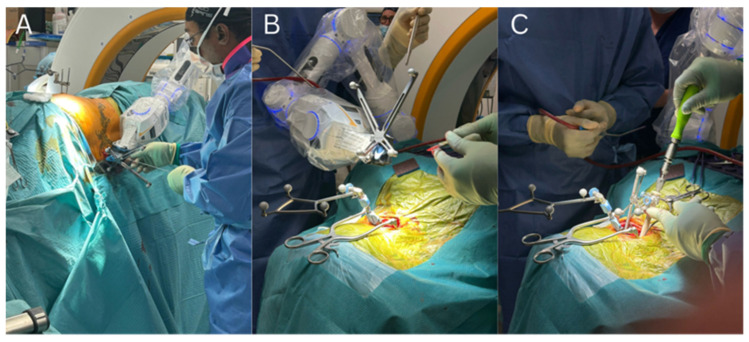
Representative operative positioning and instrumentation workflow. (**A**) Patient in the lateral decubitus position for lumbar XLIF. (**B**) Patient in the prone position for cervical robotic-assisted instrumentation. (**C**) Navigated screw placement during cervical instrumentation.

The robotic system used was the CIRQ^®^ Robotic Alignment arm (Brainlab AG, Munich, Germany), a surgeon-controlled passive arm (1.1 m maximum extension; 11 kg) mounted directly to the operating table rail and integrated with intraoperative navigation. Patients were positioned prone or lateral on a radiolucent table. A reference array was secured to either the spinous process or the iliac. Intraoperative CT (Airo, Brainlab, Munich, Germany) or pre-operative imaging was registered to the navigation system, and screw trajectories were planned at the navigation workstation. The robotic arm was then positioned over each planned entry point, with the distal module self-aligning to the predefined trajectory. A drill guide was inserted through the module, and the pilot hole was created under real-time navigation. Pedicle screws were subsequently placed using a navigated screwdriver (Figure 1A–C).

**Figure 2:**
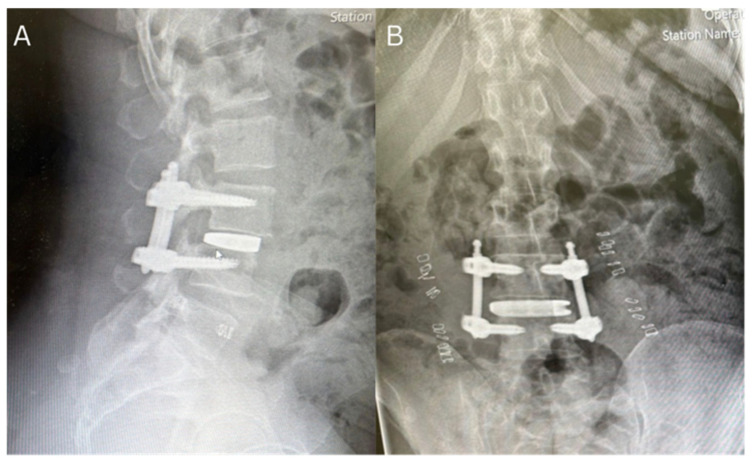
Representative post-operative plain radiographs following robotic-assisted spinal instrumentation. (**A**) Sagittal Xray of lumbar construct demonstrating screw placement and alignment. (**B**) Lumbar construct following instrumentation, confirming overall construct positioning.

Post-operative construct adequacy was confirmed by CT or plain radiographs as part of routine clinical care; the modality used was at the operating surgeon’s discretion based on clinical indication (Figure 2A,B).

### 2.4. Variables and Data Collection

Demographic variables included age, sex, body mass index (BMI), American Society of Anesthesiologists (ASA) grade, Charlson Comorbidity Index (CCI), and smoking status. Operative variables included surgical approach (open or minimally invasive (MIS)), number and level of instrumented levels (C1 to S2), operative time (incision-to-closure, hours), intraoperative radiation dose (mGy·cm^2^), number of fluoroscopy acquisitions, registration modality, and use of intraoperative neuromonitoring (IONM).

Outcome measures included length of hospital stay (LOS, derived from admission/discharge dates), time to first ambulation, robot-specific adverse events, surgical complications (neurological deficit, durotomy, screw malposition requiring revision, vascular injury, hardware failure, deep and superficial infection), 30-day readmission, and return to theatre. Patient-reported outcome measures (Oswestry Disability Index (ODI), EQ-5D) were not collected in this initial series.

Screw malposition was defined as any screw requiring intraoperative or post-operative revision for positional reasons, identified on CT or plain radiographs. All patients with available post-operative imaging (*n* = 50) had their construct reviewed by the operating surgeon for evidence of malposition. Formal Gertzbein–Robbins Scale (GRS) grading was not routinely applied, as post-operative CT is not standard clinical practice at our institution unless clinically indicated; consequently, formal blinded GRS grading was not performed. The zero-malposition figure should therefore be interpreted as a clinical revision rate. Centres employing routine post-operative CT assessment will be better positioned to report radiologically validated accuracy rates.

### 2.5. Missing Data

Missing data occurred at the variable level; all 50 patients contributed baseline and complication data. Denominators for robot complications (range 39–50) reflect the phased introduction of individual checklist fields during programme development and does not indicate patient exclusion. LOS was derived from recorded dates. Missing data were not imputed.

### 2.6. Statistical Analysis

Continuous variables were summarised as median (IQR) if non-normally distributed and as mean (SD) if normally distributed, with normality assessed using the Shapiro–Wilk test. Comparisons between elective and emergency cases were performed using the Mann–Whitney (M–W) U test for continuous variables and Fisher’s exact test for categorical variables. Wilson 95% confidence intervals were calculated for all binary complication rates.

Radiation dose and fluoroscopy events were analysed descriptively without adjustment for registration modality or surgical approach; these variables are therefore considered potential confounders rather than causal determinants and are acknowledged as a limitation.

The learning curve metrics comprised the following: (1) Spearman rank correlation between sequential case number and fluoroscopy events, operative time, and radiation dose; (2) CUSUM analysis of fluoroscopy events per case, with the potential change point identified at the maximum cumulative sum; pre- and post-change-point events were compared using the M–W U test; and (3) CUSUM analysis of operative time per instrumented level. Procedural complexity trends were assessed by comparing the number of instrumented levels in the first and last 25 cases using the M–W U test.

All tests were two-sided, with *p* < 0.05 used as a descriptive threshold. *p* values are exploratory and were not adjusted for multiplicity; findings should therefore be interpreted as hypothesis-generating rather than confirmatory. The study was not powered to detect differences in complication rates; the sample size (*n* = 50) represents the initial programme phase. All analyses were conducted in Python (version 3.10) using the verified source dataset.

## 3. Results

### 3.1. Cohort Characteristics and Spinal Range

Fifty consecutive patients underwent robotic-assisted spinal instrumentation spanning C1 to S2 during the study period. Denominators vary across analyses (range 39–50) because individual checklist and imaging fields were introduced progressively during programme development; no patients were excluded, and denominators are reported per variable throughout. The mean age was 61.9 years (SD 11.8; range 24–84), with an equal sex distribution (25 male, 25 female). Mean BMI was 29.0 (SD 5.9). ASA grade was I in 11/49 (22.4%), II in 25/49 (51.0%), and III in 13/49 (26.5%). Median CCI was 3 (IQR 2–3; *n* = 50). Current smoking status was recorded in 39 patients, of whom 13 (33.3%) were active smokers. Primary surgery was performed in 46/48 cases (95.8%); admission type was undocumented in two patients, who were therefore excluded from elective versus emergency comparisons.

Admission type was documented for 48/50 patients: 30 elective (62.5%) and 18 emergency (37.5%). Among emergency admissions: 10 (55.6%) were trauma, seven (38.9%) were degenerative acute presentations, and one (5.6%) was deformity. Among elective admissions: 28 (93.3%) were degenerative, and two (6.7%) were trauma. Overall, pathology was degenerative in 37 (74.0%), trauma in 12 (24.0%), and deformity in one (2.0%).

**Figure 3:**
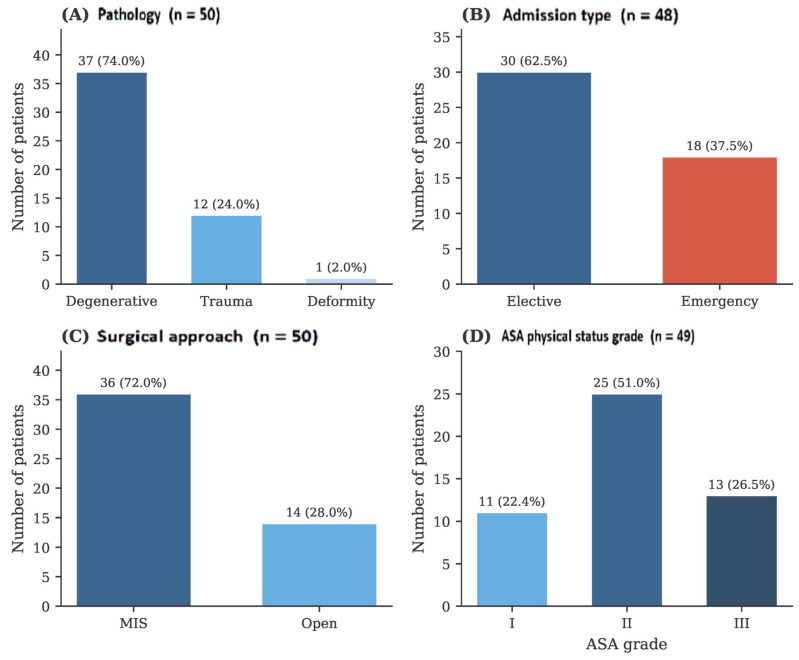
Demographic distribution: 50 consecutive, unselected patients with degenerative, traumatic, and deformity pathology. (**A**) Pathology of spinal condition, (**B**) Admission type, (**C**) Surgical approach either open or MIS (minimally invasive), (**D**) ASA of patient.

The surgical approach was MIS in 36 cases (72.0%), and open in 14 (28.0%). Registration modalities included: intraoperative CT-based navigation in 27 cases (54.0%), pre-operative CT registration in 14 (28.0%), and navigation-only in seven (14.0%), with one endoscopic and one IONM-assisted case. IONM was used in 8/41 cases (19.5%). Table 1 and Figure 3 summarise cohort characteristics.

### 3.2. Operative Metrics

The median number of instrumented levels was 2 (IQR 2–4; *n* = 44). The mean operative time, excluding case 32 (a short endoscopic procedure with robotic registration only), was 6.1 h (SD 1.7; *n* = 39). Operative time per instrumented level was a median of 2.33 h/level (IQR 1.31–3.00; *n* = 35).

Median radiation dose was 581.2 mGy·cm^2^ (IQR 85.3–1253.0; *n* = 49), corresponding to a median of 142.2 mGy·cm^2^ per instrumented level (IQR 33.7–527.0). The median fluoroscopy use was 60 acquisitions per case (IQR 12–171; *n* = 49), or 17.5 per instrumented level (IQR 3.9–56.2). Radiation dose and fluoroscopy events were not stratified by registration modality, which likely confounds radiation-related analyses and is acknowledged as a limitation.

**Figure 4:**
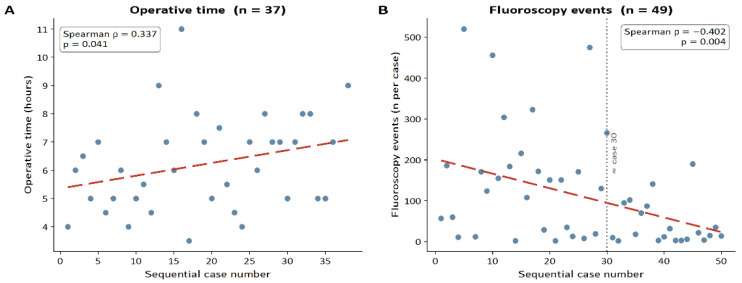
Learning curve analysis. Spearman correlations between case number and operative metrics. Fluoroscopy events: ρ = −0.402 (*p* = 0.004, *n* = 49). Operative time: ρ = −0.337 (*p* = 0.041, *n* = 37). CUSUM for fluoroscopy; median events 151 before vs. 16 after (*p* = 0.002). CUSUM for operative time/level: no clear inflection, consistent with stable efficiency. (**A**) operative time and (**B**) Fluoroscopy events.

The median LOS was 5 days (IQR 2–28) in the subset with valid date-derived records (*n* = 37; 9 excluded due to date-format entry errors). The median time to first ambulation was 4 days (IQR 2–6; *n* = 49). Distributions of operative metrics are presented in Figure 4.

### 3.3. Learning Curve Analysis

Fluoroscopy events per case demonstrated a significant negative Spearman correlation with sequential case number (ρ = −0.402, *p* = 0.004, *n* = 49), indicating a reduction in fluoroscopy use with increasing experience and suggesting improved workflow efficiency and reduced theatre staff radiation exposure over time.

CUSUM analysis suggested a potential change point in fluoroscopy events at approximately case 30, at which the cumulative sum reached its maximum. Median fluoroscopy events before this point were 151 per case (IQR 29–186; *n* = 29); compared with 16 per case thereafter (IQR 6–74; *n* = 20; M–W *p* = 0.002). This is consistent with the 20–30 case proficiency estimates reported across multiple robotic spinal platforms [7].

Operative time did not correlate significantly with case number (ρ = −0.337, *p* = 0.041, *n* = 37). CUSUM analysis of operative time per instrumented level demonstrated no clear inflection, with the cumulative sum returning towards baseline by case 50, suggesting stable rather than deteriorating overall efficiency. This should be interpreted in the context of case-mix variation: the first 25 cases had significantly higher median instrumented levels (4, IQR 2–5) compared with cases 26–50 (2, IQR 2–3; M–W *p* = 0.039), and open surgery was more common in the first half of the series (*n* = 10 vs. *n* = 4). Together, these findings suggest that stable operative times likely reflect a balance between increasing operator proficiency and variation in case complexity across the series.

**Figure 5:**
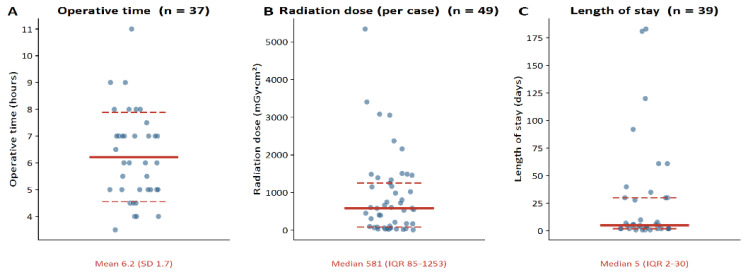
Operative metrics: instrumented levels (*n* = 44), operative time (*n* = 39), radiation dose (*n* = 49), and fluoroscopy events (*n* = 49), stratified by approach and registration modality. (**A**) Operative time, (**B**) Radiation dose, and (**C**) Length of stay.

The radiation dose did not correlate significantly with case number (ρ = +0.088, *p* = 0.547). Learning curve analyses are presented in Figure 5.

### 3.4. Complications

**Figure 6:**
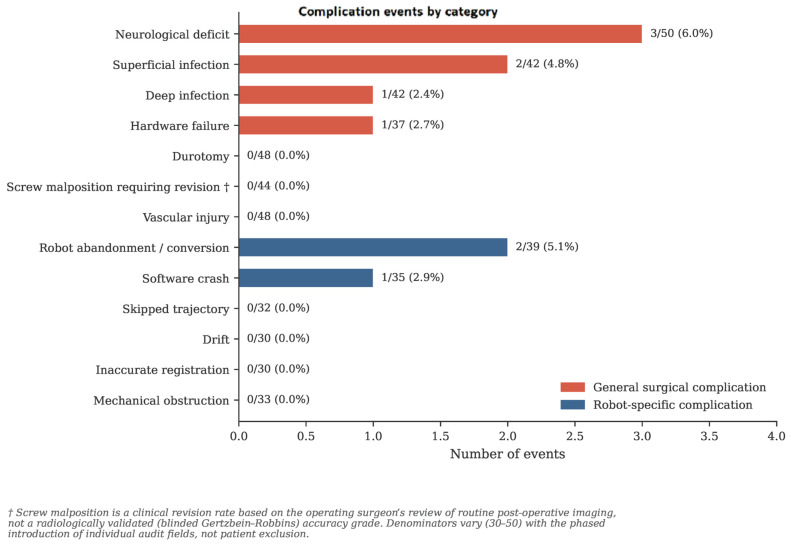
Observed complication profile with 95% Wilson CIs. Wide CIs reflect the small sample size. Zero observed rates for malposition, durotomy, and vascular injury represent clinical revision rates based on surgeon-reviewed imaging, not radiologically validated accuracy estimates.

Robot-specific adverse events are summarised in Table 2 and Figure 6. Abandonment or conversion to conventional technique occurred in 2/39 documented cases (5.1%; 95% CI 1.4–16.9%), and system malfunction in 1/48 (2.1%; 95% CI 0.4–10.9%). No events of skipped trajectory (0/49), drift (0/48), inaccurate registration (0/48), or mechanical obstruction (0/49) were recorded. Variation in denominators (39–49) reflects the phased introduction of individual checklist items during programme development and is annotated in Table 2.

Surgical complications occurred in 7/50 patients (14.0%; 95% CI 7.0–26.2%). Neurological deficits were reported in 3/50 (6.0%; 95% CI 2.1–16.2%), comprising L2 paraesthesia (*n* = 1), hand weakness (*n* = 1), and buttock pain (*n* = 1). Superficial wound infection occurred in 2/48 cases (4.2%; 95% CI 1.2–14.0%), with one case of deep infection (1/48, 2.1%) and one of hardware failure (1/48, 2.1%).

No screw malpositions requiring revision (0/49), durotomies (0/49), or vascular injuries (0/49) were identified on post-operative imaging (CT or plain radiographs) reviewed by the operating surgeon. This zero-event rate should be interpreted strictly as a clinical revision rate based on surgeon-reviewed routine imaging, not as a radiological measure of accuracy. Formal Gertzbein–Robbins Scale grading was not applied, as this is not routine clinical practice at our institution. Three patients (6.0%) were readmitted within 30 days, and two (4.0%) required return to theatre.

### 3.5. Elective Versus Emergency Comparison

**Figure 7:**
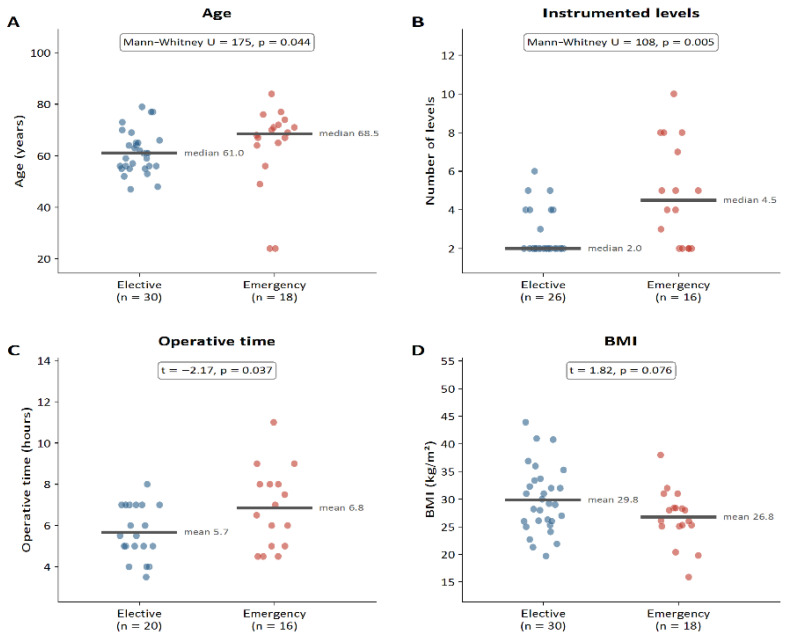
Unadjusted elective vs. emergency comparison. Emergency patients were older, more often male, required more levels, had longer operations, and underwent more open surgery. LOS, radiation, and fluoroscopy did not differ significantly. (**A**) age, (**B**) intrumented levels, (**C**) Operative time, and (**D**) BMI.

These subgroup comparisons are hypothesis-generating only and were neither prespecified nor powered for definitive inference. Emergency patients were older (median 68.5 vs. 61.0 years; *p* = 0.044), more often male (72.2% vs. 40.0%; *p* = 0.040), required more instrumented levels (median 4.5 vs. 2.0; *p* = 0.005), had longer operative times (median 7.5 vs. 5.5 h; *p* = 0.028), and were more likely to undergo open surgery (50.0% vs. 16.7%; *p* = 0.022) (Figure 7). These differences reflect the clinical characteristics of acute trauma and emergency spinal presentations rather than device-related effects.

There were no significant differences between groups in radiation dose (*p* = 0.974), fluoroscopy events (*p* = 0.106), BMI (*p* = 0.157), LOS (*p* = 0.556), or time to first ambulation (*p* = 0.213) (Table 3). All comparisons were unadjusted and should be considered exploratory.

## 4. Discussion

To our knowledge, this report presents the first published cohort study of robotic-assisted spinal instrumentation from a UK NHS unit, covering both elective and emergency cases. The existing UK robotic spine literature is limited to a single conference abstract [6], with no prior full, peer-reviewed cohort studies. In contrast, published robotic spine series to date originate predominantly from North American and European centres and are largely limited to elective lumbar degenerative pathology [6,8,9,10,11,12,13]. The present series is distinguished by its NHS setting, consecutive unselective design, and comprehensive scope, spanning the entire spinal axis (C1 to S2) across elective, emergency, trauma, degenerative, and deformity pathology.

The NHS is undergoing a significant expansion of robotic surgery, with NHS England having published its first national implementation guidance for robotic programme development in 2025 and projecting up to 500,000 robot-assisted procedures annually by 2035 [4,14]. Spinal surgery is a particularly demanding application, requiring high-precision instrumentation across the vertebral column in a heterogeneous patient population, ranging from emergency polytrauma to elective degenerative disease [2]. In this context, our experience shows that a district general neurosurgical unit with established navigation infrastructure can successfully implement a robotic spine programme across the full clinical range. The breadth of procedures—from upper cervical (C1/C2) instrumentation to sacropelvic fixation—reflects genuine clinical versatility that has not previously been characterised in a single UK series.

### 4.1. Learning Curve and Radiation Safety

CUSUM analysis suggested a potential change point in fluoroscopy events around case 30; the observed halving in median fluoroscopy events from 151 to 16 is consistent with reduced radiation exposure for theatre staff and may reflect increasing operator proficiency with the robotic workflow. This observed threshold aligns with the 20–30 case-proficiency estimates reported for multiple robotic spinal platforms by Pennington et al. [7], and represents a tangible signal achievable within the first 50 cases of a new programme. Operative time remained stable throughout the series, and the absence of a clear inflection point on CUSUM analysis for operative time per instrumented level suggests effective workflow integration without evidence of efficiency loss during programme adoption. This CUSUM finding should be regarded as hypothesis-generating rather than a validated proficiency threshold. Fluoroscopy and radiation metrics were not stratified by registration modality, surgical approach, number of instrumented levels, or pathology, each of which may influence these counts independently of operator proficiency; the apparent change point may therefore reflect evolving case mix, as well as increasing experience.

### 4.2. Emergency Robotic Spinal Surgery

The proportion of emergency cases (37.5%) in this cohort is higher than that reported in existing robotic spine series. Emergency cases showed expected differences in case mix, including older age, male predominance, a greater number of instrumented levels and open approaches, and longer operative times, reflecting the complex clinical realities of acute traumatic and degenerative spinal emergencies.

Critically, these differences did not translate into an apparent excess of robot-specific adverse events. The use of robotic and navigation-assisted instrumentation in the emergency setting may provide additional precision and safety, particularly in patients with altered or deformed anatomy, where freehand technique carries heightened risk. While CT-based neuronavigation was used for most cases in our study, MRI may provide complementary information in select cases of upper cervical trauma by exposing ligament injuries that may not be fully appreciated on CT [15]. Notably, these observations on emergency robotic spinal surgery describe operational feasibility only. Given the small, unadjusted subgroups, they should not be interpreted as evidence of equivalent safety or comparative effectiveness between the emergency and elective settings.

The present data establish the operational feasibility of robotic use in emergency spinal surgery within the NHS. However, no conclusions can be drawn regarding comparative effectiveness versus conventional fluoroscopic techniques; this will require prospective, controlled evaluation.

### 4.3. Safety Profile and Accuracy

Robot-specific adverse events were uncommon (abandonment 5.1%; malfunction 2.1%), consistent with 2–8% conversion rates in initial robotic series across platforms [11,16]. No screw malpositions requiring revision, durotomies, or vascular injuries were identified on post-operative imaging reviewed by the operating surgeon. This figure represents a clinical revision rate rather than a formal, radiologically validated screw-placement accuracy rate, as blinded Gertzbein–Robbins grading and routine post-operative CT review were not performed.

Published robotic series with formal GRS grading have reported 92.9–95.9% Grade A screw rates [10,12]; our data are consistent with these benchmarks but cannot confirm them without equivalent blinded radiological assessment. Direct comparison with published robotic series is limited by heterogeneous pathology mixes and accuracy definitions; however, our overall complication and robot-specific event rates fall within the ranges reported in early phase robotic cohorts. The overall 14% surgical complication rate should be interpreted in the context of the unselected cohort including 37.5% emergency admissions and a median CCI of 3; it does not implicate the robotic system and is broadly consistent with the complexity of the caseload managed.

### 4.4. Limitations

This study has several important limitations. First, it is a single-centre feasibility series (*n* = 50), and the findings are not generalisable; the sample size is insufficient to support definitive conclusions about safety or efficiency. The centre has since performed over 70 robotic procedures, and a subsequent analysis will be reported separately.

Second, there was no concurrent control group; therefore, outcomes cannot be attributed specifically to the robotic platform rather than to surgeon experience, existing navigation infrastructure, or case selection. Third, selection bias is possible, as cases performed without navigation—due to system unavailability or immediate surgical urgency—were not captured. If such cases differed systematically in complexity or risk profile, safety estimates may be biased; however, the direction and magnitude of this bias cannot be determined from the present dataset.

Fourth, missing operative time data (10/50), concentrated in earlier cases, may have influenced learning curve analyses. Fifth, formal Gertzbein–Robbins Scale (GRS) grading of screw accuracy was not performed; the reported zero-malposition rate reflects a clinical revision rate based on surgeon-reviewed imaging rather than a radiologically validated accuracy measure. Future analyses will incorporate systematic, blinded postoperative CT assessment.

Sixth, patient-reported outcome measures (ODI, EQ-5D-5L) were not collected, representing a key limitation in assessing clinical impact; this is being addressed in the next phase of the programme. Seventh, radiation dose and fluoroscopy metrics were not stratified by registration modality, which is likely to be a significant confounder in radiation-related analyses. Finally, multiple exploratory comparisons were performed without adjustment for multiplicity, increasing the likelihood of type I error; accordingly, no individual finding should be considered confirmatory.

### 4.5. Future Directions

Ongoing prespecified objectives include prospective CUSUM monitoring from case 51 onwards; systematic, independent, blinded post-operative CT assessment with GRS grading; PROMs collection (ODI, EQ-5D-5L); concurrent navigation-only matched-case comparison; characterisation of robotics across spinal oncology and revision surgery and more comprehensive documentation of complications, to include symptom onset, severity, duration and recovery. These observations will form the basis of the programme’s second published report. Future work will ultimately require multi-centre prospective registries to validate learning curves and complication rates across diverse NHS units adopting robotic spine platforms, given the infrastructure and case-mix variation inherent in the UK healthcare system. Future studies should also evaluate pathology specific applications of robotic spinal instrumentation, including upper cervical trauma and hangman’s fractures, to define its role, along with advanced imaging in more technically demanding cases [17].

## 5. Conclusions

To our knowledge, this is the first published UK NHS cohort study of robotic spinal instrumentation that includes both elective and emergency cases across the full spinal axis from C1 to S2. Robotic-assisted instrumentation was feasible across this diverse, unselected caseload, with a low observed complication rate. CUSUM analysis suggested a potential change point in fluoroscopy events around case 30, consistent with published robotic spine proficiency thresholds, and with a reduction in theatre staff radiation exposure as experience with the robotic workflow accumulated. No screw malpositions requiring revision were identified on surgeon-reviewed post-operative imaging; formal radiological accuracy assessment was not performed, as post-operative CT grading is not standard clinical practice at our institution. As the NHS expands its robotic surgery programme, this series provides an early evidence base for robotic spinal instrumentation across the breadth of pathology encountered in a UK tertiary neurosurgical centre. These findings support feasibility across the elective and emergency caseload; they do not establish comparative effectiveness or equivalent safety between these settings, nor a radiologically validated measure of screw accuracy. Larger comparative studies with formal accuracy assessment are required.

## Figures and Tables

**Table 1:** Cohort characteristics (*n* = 50).

Variable	Value/Frequency	Available *n*
Mean age, years (SD; range)	61.9 (11.8; 24–84)	50
Sex—Male/Female, *n* (%)	25/25 (50%/50%)	50
Mean BMI (SD)	29.0 (5.9)	50
ASA I/II/III, *n* (%)	11 (22.4%)/25 (51.0%)/13 (26.5%)	49
Median CCI (IQR)	3 (2–3)	49
Current smoker, *n* (%)	13 (33.3%)	39
Primary surgery, *n* (%)	46 (95.8%)	48
Admission—Elective/Emergency, *n* (%) ^†^	30 (62.5%)/18 (37.5%)	48
Pathology—Degenerative/Trauma/Deformity, *n* (%)	37 (74.0%)/12 (24.0%)/1 (2.0%)	50
Emergency pathology—Trauma/Degenerative/Deformity, *n* (%)	10 (55.6%)/7 (38.9%)/1 (5.6%)	18
Approach—MIS/Open, *n* (%)	36 (72.0%)/14 (28.0%)	50
Registration—Intraop CT + Nav/Pre-op CT/Nav-only, *n* (%)	27 (54.0%)/14 (28.0%)/7 (14.0%)	50
IONM used, *n* (%)	8 (19.5%)	41

^†^ 2 patients had undocumented admission type and were excluded from the elective/emergency breakdown. All denominators reflect available data; missing data were not imputed. BMI = body mass index; ASA = American Society of Anesthesiologists; CCI = Charlson Comorbidity Index; MIS = minimally invasive surgery; Nav = intraoperative navigation; IONM = intraoperative neuromonitoring.

**Table 2:** Observed complications with 95% Wilson confidence intervals.

Complication	*n*/Denominator ^†^	Rate (%)	95% CI (%)
**Robot–specified adverse events**			
Abandonment/conversion to conventional	2/39	5.1	1.4–16.9
System malfunction	1/48	2.1	0.4–10.9
Skipped trajectory	0/49	0	0–7.2
Drift	0/48	0	0–7.4
Inaccurate registration	0/48	0	0–7.4
Mechanical obstruction	0/49	0	0–7.2
**Surgical complications**			
Any surgical complication	7/50	14.0	7.0–26.2
Neurological deficit ‡	3/50	6.0	2.1–16.2
Superficial wound infection	2/48	4.2	1.2–14.0
Deep infection	1/48	2.1	0.4–10.9
Hardware failure	1/48	2.1	0.4–10.9
Durotomy	0/49	0	0–7.2
Screw malposition requiring revision §	0/49	0	0–7.2
Vascular injury	0/49	0	0–7.2
**Post-operative**			
30-day readmission	3/50	6.0	2.1–16.2
Return to theatre	2/50	4.0	1.1–13.5

^†^ Robot-specific denominators (39–49) reflect the phased introduction of individual checklist fields during programme development, not patient exclusion. ‡ Neurological deficits by free text: L2 paraesthesia (*n* = 1), hand weakness (*n* = 1), buttock pain (*n* = 1); none attributable to screw malposition. § Screw malposition was assessed by the operating surgeon’s review of post-operative imaging (CT or plain radiographs, available for *n* = 49); formal Gertzbein–Robbins Scale grading was not applied, as this is not routine clinical practice at this institution. This figure represents a clinical revision rate.

**Table 3:** Elective versus emergency comparison (unadjusted; exploratory).

Variable	Elective (*n* = 30)	Emergency (*n* = 18)	*p*-Value
Median age (IQR), years	61.0 (56.0–65.0)	68.5 (64.2–71.8)	0.044 *
Male sex, *n* (%)	12 (40.0%)	13 (72.2%)	0.040 *
Median BMI (IQR)	29.1 (26.0–33.1)	27.1 (25.3–28.4)	0.157
Median levels instrumented (IQR)	2.0 (2.0–3.8)	4.5 (2.0–7.2)	0.005 **
Median operative time (IQR), hours †	5.5 (5.0–7.0)	7.5 (5.8–8.2)	0.028 *
Open approach, *n* (%)	5 (16.7%)	9 (50.0%)	0.022 *
Median radiation dose (IQR), mGy·cm^2^	546.4 (68.8–1253.0)	565.6 (140.7–1118.7)	0.974
Median LOS (IQR), days	5.0 (2.0–30.0)	5.0 (3.5–9.0)	0.556

Mann–Whitney U for continuous variables; Fisher’s exact for sex and approach. † Operative time available for 26 elective and 13 emergency patients. LOS from date-derived records (*n* = 37 valid). * *p* < 0.05; ** *p* < 0.01. No multiplicity correction applied. Sample sizes are small; results should not be used to draw clinical inferences about emergency versus elective outcomes without a comparative control group.

## Data Availability

The data presented in this study are available on request from the corresponding author. Access is restricted because the data were collected as part of a clinical governance audit under BHRUT R&D oversight and are subject to NHS ethical and information governance requirements.

## Abbreviations

The following abbreviations are used in this manuscript:

NHS	National Health Service
UK	United Kingdom
BHRUT	Barking, Havering and Redbridge University Hospitals
R&D	Research and Development
STROBE	STrengthening the Reporting of OBservational studies in Epidemiology
CUSUM	Cumulative sum
CT	Computed tomography
BMI	Body mass index
ASA	American Society of Anesthesiologists
CCI	Charlson Comorbidity Index
MIS	Minimally invasive surgery
IONM	Intraoperative neuromonitoring
LOS	Length of hospital stay
ODI	Oswestry Disability Index
EQ-5D	EuroQol five-dimension questionnaire
EQ-5D-5L	EuroQol five-dimension five-level questionnaire
GRS	Gertzbein–Robbins Scale
IQR	Interquartile range
SD	Standard deviation
M–W	Mann–Whitney
CI	Confidence interval
PROMs	Patient-reported outcome measures
C1	First cervical vertebra
C2	Second cervical vertebra
S2	Second sacral vertebra
mGy·cm^2^	Milligray square centimetres
